# Bird diversity along elevational gradients in the Dry Tropical Andes of northern Chile: The potential role of Aymara indigenous traditional agriculture

**DOI:** 10.1371/journal.pone.0207544

**Published:** 2018-12-05

**Authors:** Paola Araneda, Walter Sielfeld, Cristián Bonacic, José Tomás Ibarra

**Affiliations:** 1 Fauna Australis Wildlife Laboratory, Department of Ecosystems and the Environment, School of Agriculture and Forest Sciences, Pontificia Universidad Católica de Chile, Santiago, Chile; 2 Centro de Investigación en Medio Ambiente (CENIMA), Universidad Arturo Prat, Región de Tarapacá, Chile; 3 *ECOS* (*Ecology-Complexity-Society*) Laboratory, Centre for Local Development, Education and Interculturality (CEDEL), Villarrica Campus, Pontificia Universidad Católica de Chile, Villarrica, Región de La Araucanía, Chile; 4 Centre for Intercultural and Indigenous Research (CIIR), Faculty of Social Sciences, Pontificia Universidad Católica de Chile, Santiago, Chile; 5 Millennium Nucleus Centre for Socioeconomic Impact of Environmental Policies (CESIEP), Pontificia Universidad Católica de Chile, Santiago, Chile; University of South Carolina, UNITED STATES

## Abstract

Understanding diversity patterns along environmental gradients lies at the heart of community ecology and conservation. Previous studies have found variation in bird diversity and density along “natural” elevational gradients in the Tropical Andes Hotspot. However, there is still a lack of knowledge about how bird communities respond to traditional land-use patterns, in association with other multiple drivers, along elevations. In the present study, we investigated biotic, abiotic and anthropogenic sources of variation associated with bird species diversity, density and turnover along a 3000-m elevational gradient, in southern limit of the Tropical Andes Hotspot, northern Chile. Over four seasons, we conducted 472 bird point count surveys and established 118 plots distributed across the Desert, Pre-Puna, Puna and High-Andean belts, where biotic, abiotic and anthropogenic factors were measured. We used mixed-effects models to estimate alpha diversity and multinomial Poisson mixture models to estimate species density, accounting for detectability. Species diversity and density increased until 3300 masl and then declined. This type of elevational pattern is characteristic of dry-based mountains, where environmental conditions are suitable at mid-elevations. Habitats shaped by traditional Aymara indigenous agriculture, associated with relatively high vegetation heterogeneity, hosted the highest values of bird diversity and density. Species turnover was structured by habitat type, while elevational ranges of most species were restricted to three relatively discrete assemblages that replaced each other along the gradient. Our study revealed a hump-shaped relationship between elevation and bird diversity and density in the Dry Tropical Andes Biodiversity Hotspot, supporting a diversity pattern characteristic of dry-based mountains of the world. Traditional Aymara agriculture may have constructed ecological niches for biodiversity at mid-elevations, enhancing vegetation heterogeneity, thus providing resources for resident and rare species. Increasing loss of traditional land-use may present a threat to the bird community in the Tropical Andes Hotspot.

## Introduction

Montane zones are typically rugged landscapes uplifted to such a level that local climate is affected [1]. Mountains host exceptional biodiversity due to the elevational gradient that results from the variation of climate and topography over distances of only a few kilometers [2]. These ecological transition areas (ecotones) are often characterized by high species turnover rates or beta diversity [3]. Furthermore, mountains have been subject to human land-use practices for millennia and, as a result, harbor the largest number of distinct ethnic groups, varied remnants of biocultural traditions, and human-habitat adaptations through agriculture [4,5]. General diversity patterns along elevational gradients are the result of the combined effects of complex, often nonlinear, processes that show covariation with elevation [6]. Conventionally, species richness has been considered to decrease monotonically with increasing elevation, while the elevational ranges of some species are greater at high elevations than at low elevations. This is the so-called “Stevens’s rule” [7]. However, Rahbek [8,9] rejected Stevens’s rule by showing that many elevational gradients have mid-elevation peaks in diversity.

Climatic variation can influence the composition of bird communities along elevational gradients [10,11]. In temperate regions, birds are sensitive to seasonality due to both resource bottlenecks for food and water availability, and temperature regulation requirements across seasons [12,13]. In arid mountain ecosystems, maximum bird diversity can occur in wetter and cooler climatic conditions, generally at mid-elevations [14,15]. Vegetation heterogeneity (e.g. vegetation strata with dense foliage) is often correlated with bird species richness at various geographical scales [6,16,17]. For example, mid-elevation habitats with high vegetation heterogeneity in the Eastern Himalaya influence peaks in species diversity and density along the elevational gradient [18]. Furthermore, relatively high habitat diversity along elevational gradients may harbor many co-existing species within habitat types, resulting in high species turnover between different habitats [19,20].

Through various land-use practices, humans have shaped almost every corner of the Earth, and thus influence the diversity and structure of ecological communities [21]. Within mainstream ecological literature, humans are generally treated as exogenous drivers of change [22]. However, relatively recently, researchers have expanded this mainstream notion to identify humans as multidirectional participants in coupled social-ecological systems [23]. For example, through traditional agricultural practices, such as terracing and ridged crop systems in the Andes, humans have for millennia constructed ecological niches for biodiversity in mountain areas [5,24–26]. However, the role of traditional agriculture, in association with multiple other drivers (e.g. climate and vegetation) along elevational gradients, has not been subject to detailed empirical assessment in bird community ecology studies [6,27].

The Tropical Andes Hotspot is the most diverse hotspot on Earth, with higher numbers of species and rates of endemism than any other [28,29]. The hotspot contains a high variety of habitat types resulting from steep altitudinal gradients and climatic factors caused by the interaction of complex topography, trade winds, oceanic influences [30] and, potentially, indigenous use of Andean slopes for agriculture over the course of millennia [31,32]. This variety of habitat types may host bird species with narrow environmental tolerances, resulting in limited species distributions along elevational gradients [1,7]. It may be expected that this pattern will relate to rapid turnover–or beta diversity–along the elevational gradient, particularly when local diversity–or alpha diversity–is a small fraction of the total landscape diversity [19].

Studies conducted on birds along elevational gradients in the Wet Tropical Andes (Peru, Colombia and Bolivia) have shown a decline in species richness with elevation, due to a decrease in temperature and vegetation cover, and proximity to human settlements [15,33–36]. By contrast, Kessler et al [37], found a unimodal pattern relationship between richness and elevation in the forests of the Bolivian Andes, with a peak in bird species richness associated with the presence of old-growth forests at intermediate elevations (2700–3150 masl). The Dry Tropical Andes Region, which includes northern Argentina and Chile, is still relatively unexplored, and most of the available literature is descriptive [38–42]. This is especially true of the southern limit of the Tropical Andes Hotspot, which includes a complex of mountain chains and valleys, bordered to the south by the extremely arid Atacama Desert [30,43].

In the present study, we examined bird diversity and density patterns along an elevational gradient in the Dry Tropical Andes of northern Chile. We then evaluated a suite of biotic and abiotic factors that may be correlated with bird diversity and density, focusing on climatic conditions, seasonality, elevation, vegetation heterogeneity and habitat type, and giving special attention to the association between indigenous traditional agriculture and bird communities along the elevational gradient. Finally, we assessed the turnover (beta diversity) and range patterns of each bird species along the elevational gradient. We predicted that (1) vegetation heterogeneity drives non-linear associations between elevation and diversity and density, and (2) habitat diversity along the gradient can host different bird assemblages, resulting in high species turnover and distinct communities. To test these predictions, we estimated alpha diversity, density and beta diversity along a gradient of 3000 meters of elevation. This gradient ranged from Desert belt through Pre-Puna–with semi-arid vegetation and indigenous Aymara agriculture–to Puna and the High Andean belt above 4000 meters of elevation.

## Methods

### Study area

**Fig 1:**
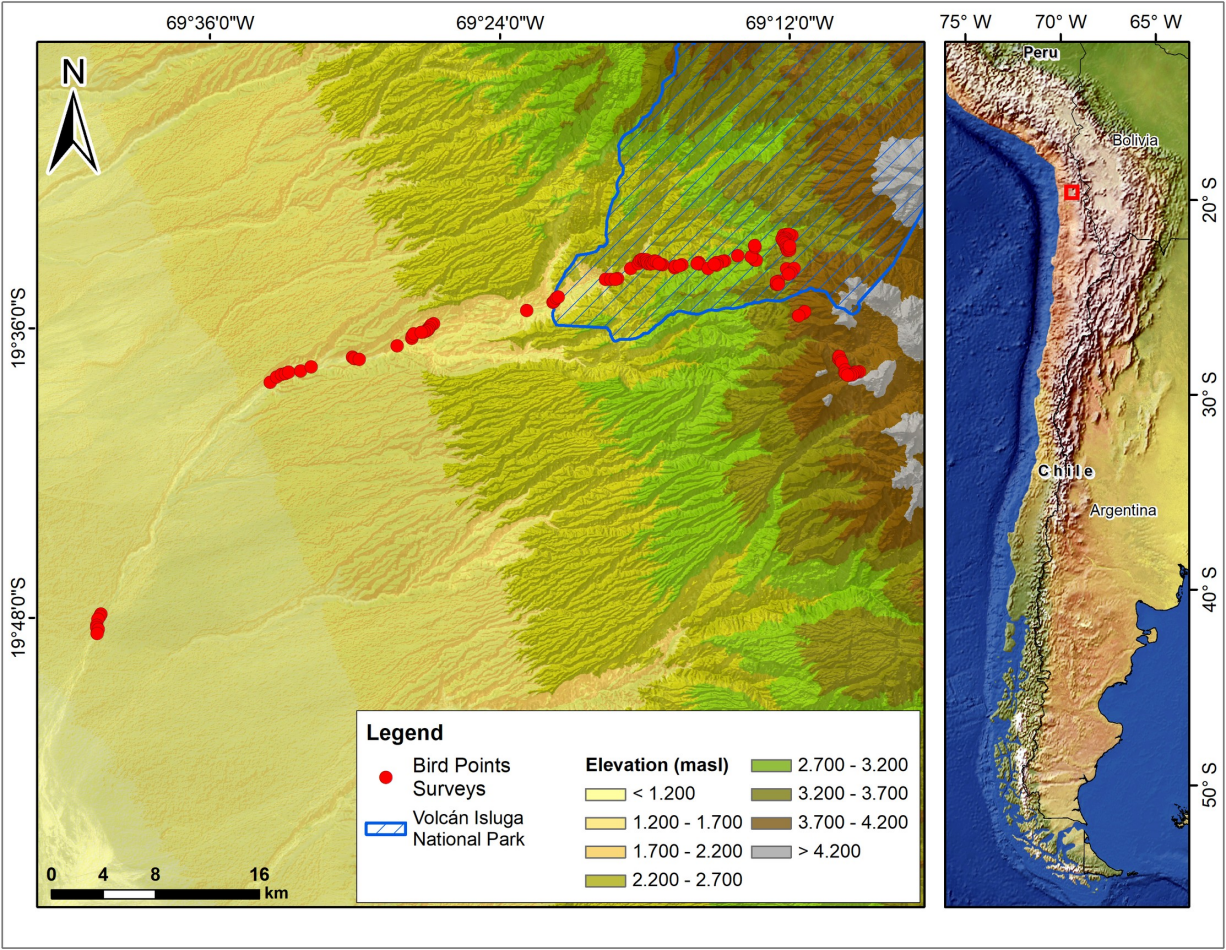
Study area showing surveyed points along an elevational gradient in the Aroma/Chiapa basin, Dry Tropical Andes of Northern Chile. Circles show the 118 surveyed points, and the striped polygon indicates the western boundary of the Volcán Isluga National Park. Source: Base map from Natural Earth.

The study was conducted in the Aroma/Chiapa basin, located on the western limit of the Volcán Isluga National Park, in the Dry Tropical Andes of northern Chile (Fig 1). Aroma/Chiapa is an east-west drainage basin with a length of 70 km, encompassing part of the Desert, Pre-Puna, Puna and High-Andean belts [43,44]. The area receives rainwater during the summer season (45.56 ± 35.32 mm), mainly between January and March when the Inter-tropical Convergence Zone (ITCZ) moves to the south [43,45]. The basin has almost continuous surface runoff, and its tributaries are of an ephemeral regime [46]. The surveyed points ranged from 1200, where basin starts, (19°47’53”S 69°40’36”W) to 4120 m above sea level, where basin ends (masl; 19°38’2”S 69°9’19”W).

Vegetation cover varies with elevation, and vegetation formations have been described as distinct belts associated with different elevations [47]. Luebert and Pliscoff [43] and Trivelli and Valdivia [44] proposed the following classifications. (1) Desert belt (<2500 masl), where it is possible to find the Inner Tropical Desert Formation, with sparse vegetation and dominance of *Tessaria absinthioide*s and *Ditichlis spicata*. The Inner Tropical Low Desert Scrub Formation–an open and xeromorphic scrubland dominated by *Adesmia atacamensis* and *Cistanthe celosioides*–is also present in the Desert belt. In addition, it is possible to find intrazonal vegetation associated with streams, such as *Pleocarphus revolutus*, *T*. *absinthioide*s and *Cortaderia atacamensis*. (2) Pre-Puna belt (2500–3200 masl), characterized by an Andean Tropical Low Desert Scrub Formation: a very open scrub, with or without succulents, generally dominated by *Atriplex imbricata* and *Acantolipia deserticola*. The Pre-Puna belt also comprises an Andean Tropical Spiny Forest Formation, which is a sparse, extremely xeromorphic forest, dominated by Cactaceae *Browningia candelaris* and *Corryocactus brevistylus* in the upper canopy, with a low shrub stratum of succulents. Riparian vegetation is represented chiefly by *C*. *atacamensis* and some Fabaceae trees. In this belt, it is also very common to find patches of traditional Aymara agriculture [48]. Aymara agriculture chiefly comprises subsistence farming of a diverse set of traditional crops, such as maize, alfalfa, potatoes, oregano and banana passionfruit (*Passiflora mollissima*). This traditional land-use includes a water irrigation mechanism in the form of crop terraces [24,48]. Aymara agriculture has likely been conducted in the area for around 1100 years, and remains a system of communal ownership and management of the streams that descend from the *Tata’Jachura* volcano [49–51]. (3) The Puna belt (3200–4000 masl) is characterized by an Andean Tropical Low Scrub Formation of zonal vegetation, comprising a dense thicket dominated by *Fabiana ramulosa*, *Diplostephium meyenii*, *Lophopappus tarapacanus* and *Baccharis boliviensis* in the woody strata, which can reach 1m in height. Some Cactaceae such as *C*. *brevistylus* are also present in this belt. (4) The High-Andean belt (>4000 masl) is represented by the Andean Tropical Low Shrub Formation of zonal vegetation dominated by *Parastrephia lepidophylla* and *P*. *quadrangularis*, where abundant *Festuca orthophylla* and *Tetraglochin cristatum* can also be found. No intrazonal vegetation was found in the Puna or High-Andean belts.

### Avian surveys

We randomly established 118-point surveys, each at a distance of at least 150m from adjacent points. These points were grouped into 19 elevational intervals, generated by grouping three to seven survey points (6.21 ± 0.34) for every 150m of elevation. We surveyed each point twice during both the wet (February and April) and dry (November and May) seasons of 2016 and 2017 (n = 472). Each point survey lasted 6 minutes, during which every bird seen or heard within a 50m radius was recorded. The distance to each bird was estimated and grouped into two distance intervals (0–25 and 26-50m) for further analysis. Birds were recorded during the four hours of peak activity immediately after dawn. In each point survey we recorded temperature (°C), humidity (%) and wind speed (m/s) using a handheld weather monitor (WM-300 WindMate, Speedtech Instruments, USA; Table 1) [52,53].

**Table 1:** Candidate covariates for detectability and density estimations used in the analysis.

Type of covariate (abbreviation)	Description
** 1. Temporal and weather covariates for detectability**	
**1.1 Season (SEA)^a^**	1: wet season; 2: dry season.
**1.2 Time (TIM)**	Time of survey (minutes since 06:30)
**1.3 Date (DAT)**	Julian date
**1.4 Noise (NOI)**	Environmental noise. 0: quiet; 1: substantial (wind noise, and/or river and stream noise)
**1.5 Temperature (TEM)**	Temperature (°C)
**1.6 Humidity (HUM)**	Relative Humidity (5% to 95%)
**1.7 Wind speed (WIN)**	Average wind (m/s), over 10 seconds
** 2. Environmental covariates for diversity and density**	
**2.1 Habitat type (HAT)**	50 m radius plot. 1: desert habitat; 2: arboreal shrubland habitat; 3: habitat of columnar cactus; 4: agricultural habitat; 5: highland steppe habitat; 6: riparian habitat
**2.2 Vegetation Complexity (COM)**	Number of vegetation strata. Herbaceous stratum: 0–1 m; Low Woody stratum: ≤0.5 m; Medium Woody stratum: 0.5–1; High Woody stratum 1–2 m; Arboreal stratum: ≤3m; High Arboreal stratum > 3 m
**2.3 Vegetation Heterogeneity (HET)**	Sum of the foliage coverage index of the vegetation strata. 0: absence of stratum; 1: ≤5% coverage; 2: 5%-25%; 3: 25%-50%; 4: 50%-75%; 5: 75%-95%; 6: 95%-100%.
**2.4 Elevation (ELE)**	Meters above sea level measured at the center of the plot

^a^ Wet season: December, January, February, March, April. Dry season: May, June, July, August, September, October, November.

### Habitat measures

We used previous studies of bird-habitat relationships along mountain elevational gradients to identify potential structural habitat attributes (hereafter covariates) that may influence distribution patterns of birds in the Andes [33,54–56]. We located habitat plots (50m radius; 0.79 ha; n = 118 plots) at the center of the previously described point survey. Each plot (point survey) was assigned to a habitat type according to the seven criteria described in Table 1. We then characterized the habitat structure at each plot [41,47,57] and estimated vegetation heterogeneity for six vertical strata based on a six-point scale (Table 1). Our definition of vegetation heterogeneity states that heterogeneous plots have many vegetation strata with dense foliage coverage [58,59]. Therefore, vegetation heterogeneity was estimated by summing the coverage index of each vegetation stratum (Table 1).

### Data analysis

#### Bird diversity and composition

We estimated alpha diversity using Generalized Linear Mixed-Effect (GLME) models with a Poisson type error [60], using lme4 [61] and AICcmodavg packages [62] in R [63]. GLMEs describes the relationship between a response variable and several explanatory covariates (fixed effects) collected in aggregated units at different levels (random effects). We tested the fixed effect of habitat type, elevation, heterogeneity and seasonality on bird richness. Elevational interval, year, elevational interval-by-heterogeneity and seasonality-by-heterogeneity interaction were used as random effects (Table 1). The strength of evidence of fifteen models was evaluated by calculating model weights (*w*_*i*_) and the AIC value [60,64]. Models with AIC < 2 were considered to be just supported by the data [65].

Species richness was defined as the total number of recorded species by point survey. While, alpha diversity was the estimated number of species for each point survey by the GLME. We calculated beta diversity by the dissimilarity in presence and absence of species composition comparing neighboring intervals, utilizing the Sørensen’s Index of Dissimilarity Sør = 2*a*/(2*a* + *b* + *c*), where *a* is the number of species common between two points, *b* the number of species unique to first point, and *c* the number of species unique to the second point [66]. Sørensen’s index is dependent on variation in the matching component *a*, or the level of continuity in species composition between two points [67].

#### Bird detectability and density

Bird density estimates will vary according to species detectability, which may be influenced by the distance of the recorded bird from the observer and other survey-specific covariates, including temporal factors and weather conditions [68,69]. The Multiple-Covariate Distance Sampling (MCDS) framework uses the observer distance distribution, *y*, and one or more additional covariates represented by the vector z, to model the detection function. Therefore, the probability of detection is denoted as *g*(*y*, z) [70].

Using MCDS we analyzed avian point surveys utilizing Multinomial Poisson Mixture Models [68]. To estimate detection (*p*) and density (*D*) for each species across points, we used maximum-likelihood methods in the R-Unmarked [71,72] program from [63]. To model *D*, we first assessed collinearity to reduce the number of covariates. Collinear covariates were removed (r > 0.7), maintaining only the ones predicted to be more biologically influential for each species [41,54,57]. The half-normal key function for the detection function was selected using Akaike's Information Criterion (AIC) [64,70]. Detectability (*p*) was estimated using eight covariates potentially affecting the scale parameter of the detection function: season, time, date, noise, temperature, humidity and wind speed (Table 1). Important covariates for each species were identified by AIC [73].

After correcting for *p* of each species, we estimated density (individuals per hectare) by taking habitat type (HAB), vegetation heterogeneity (HET) and elevation (ELE) as covariates. To obtain the best model for each species, we computed model weights (*w*_*i*_) and the AIC value, following the same rationale described above. Once a preferred approach was selected, we averaged the density estimates from competing models (ΔAIC <2) [65]. For analyses of detectability and density, we used a subset of the most abundant species. For both alpha diversity and density, a Kruskal-Wallis with Wilcoxon post-hoc multiple pairwise tests was used to examine differences between habitat types. We also performed linear and nonlinear regression to fit models of bird diversity and density on vegetation heterogeneity and elevation, and ranked them according to Akaike’s information criterion adjusted for small samples (AICc) [65].

We obtained research permits from the Chilean National Park administration authority (CONAF number 194195) to work in the Volcán Isluga National Park, and consent from the “*Tata’Jachura*” Chiapa Aymara Indigenous Community.

## Results

### Species composition and elevational range size

We recorded 49 bird species across 118 survey points along the elevational radient from 1200 to 4120 masl (See S1 Appendix). Seven bird orders were recorded, which included 14 families. Tyrannidae and Thraupidae were the most represented families, each with nine species. Furnariidae followed these families with eight species, then Columbidae with five and Trochilidae with four.

**Fig 2:**
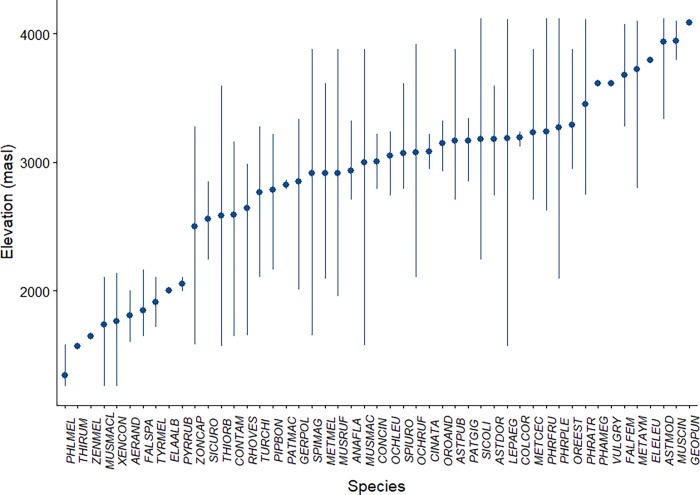
Elevational range sizes for 49 species occurring along an altitudinal gradient in the Dry Tropical Andes of northern Chile, between November 2016 and August 2017. Lines indicate the maximum and minimum elevational limits of each species range, and species are ordered along the abscissa by ranked elevational midpoints (range average). See “S1 Appendix” for codes of bird species.

Elevational ranges showed that three relatively discrete assemblages of birds replaced one another along the elevational gradient (Fig 2). Only three species–*Metropelia aymara*, *Falco femoralis* and *Asthenes modesta*–were recorded across a broad range of elevations. Mid-elevation habitats in the Pre-Puna belt showed the largest number of species (2500–3300 masl). Here, 61.2% (30) of total bird species were found, being mainly Passeriformes, Apodiformes and Columbiformes. A total of 18 species were exclusive to the Pre-Puna belt, such as *Patagioenas maculosa* and *Colibri coruscans*, whereas 12 species had a broader elevational range and were also present in other belts, such as *Lepthastenura aegitaloides* and *Muscisaxicola maculirostris*. A total of 10 species (20.4%) were registered exclusively in the lowlands of the Desert belt (<2500 masl), such as *Aeronautes andecolus* and *Xenospingus concolor*. Finally, Passeriformes and Falconiformes accounted for the majority (both with four species) of the nine species (18.4%) found in the Puna and High-Andean belts.

Elevational ranges varied among species. Then, 19 (38.8%) of the species showed elevational ranges of 500 m, and 30 (61.2%) had a range of more than 1000 m. Only one species (*L*. *aegitaloides*) showed an elevational range of 3000 m, covering the entire study area. Six species were detected only at a single elevation point.

### Alpha diversity

The medians of alpha diversity between elevational intervals were significantly different (Kruskal-Wallis test; H = 415.3, d.f. = 18, *p*<0.05). For GLME analyses, the model including habitat type (HAB), heterogeneity (HET), elevation (ELE) and seasonality (SEA) as fixed effects performed better than all the other models (Table 2).

**Table 2:** Top five ranking models used to estimate the effect of environmental covariates^(a)^ on species richness along an elevational gradient in the Dry Tropical Andes of northern Chile, between November 2016 and August 2017. The models are ranked in descending order according to AICc.

Model	n	K	AICc	Δ_AICc	AICcWt	Cum.Wt	LL
**HAB+HET+ELE+SEA+** **(HET/INT)**	473	12	1365.99	0	0.76	0.76	-670.66
**HAB+HET+ELE+SEA+** **(INT)+(SAM)+(HET/INT)**	473	14	1368.65	2.66	0.2	0.97	-669.87
**HAB+HET+ELE+SEA+** **(INT)+(SAM)**	473	11	1373.62	7.63	0.02	0.98	-675.52
**HAB+HET+ELE+SEA+** **(INT)**	473	10	1373.75	7.76	0.02	1	-676.64
**HAB+HET+ELE+SEA+** **(INT)+(SAM) + (HET/SEA)**	473	14	1379.46	13.47	0	1	-675.27

^(a)^Model covariates: HAB: habitat type; HET: heterogeneity; ELE: elevation; SEA: season; INT: elevational intervals; SAM: year when surveys were conducted.

n: sample size; K: number of parameters; AICc: value according to Akaike’s Information Criterion corrected for small samples Δ_AICc: the difference in Akaike’s Information Criterion corrected for small sample sizes; AICcWt: Akaike weight; Cum.Wt: accumulated weight of the Akaike value; LL: likelihood.

**Fig 3:**
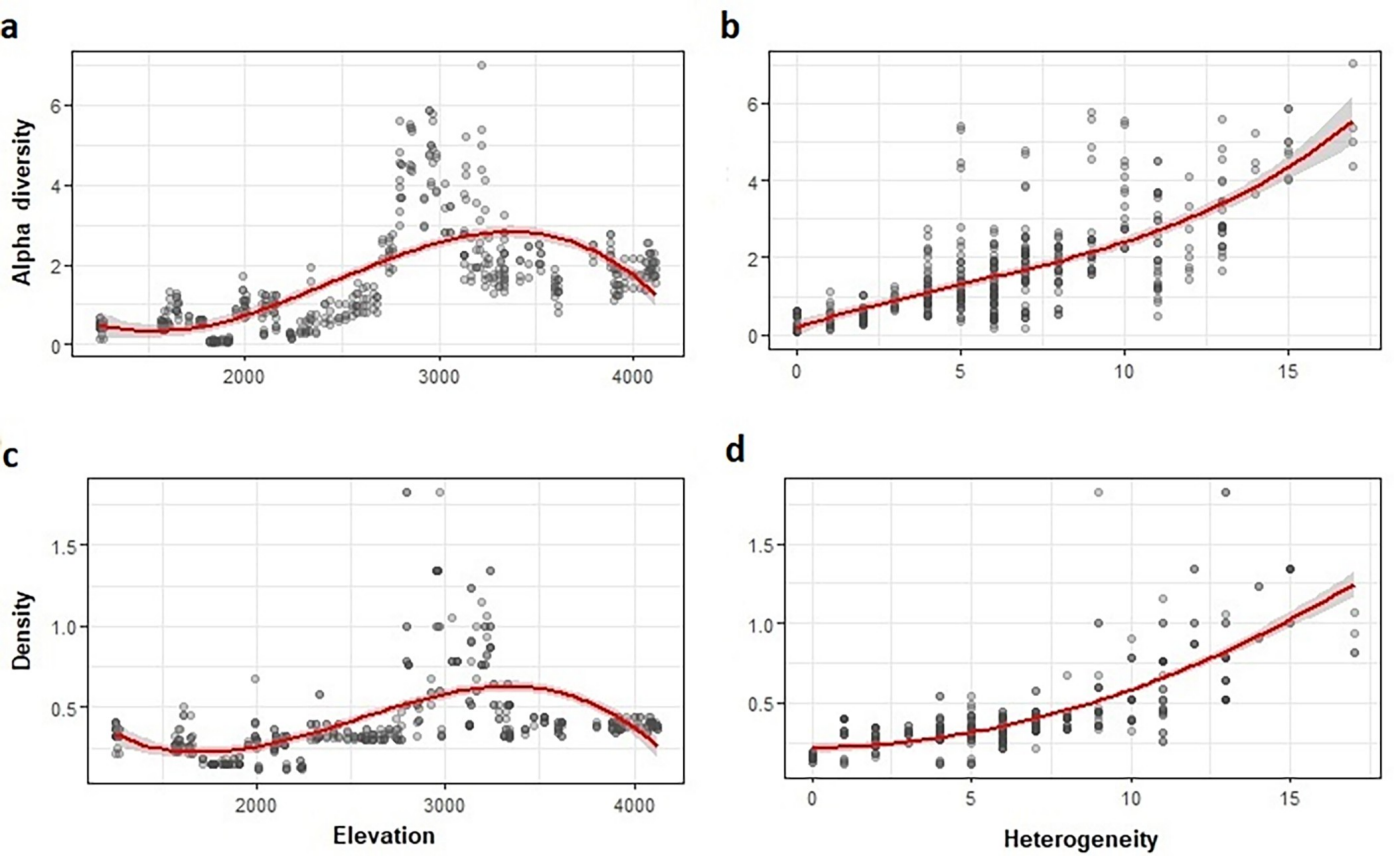
**Relationship between (a) elevation and alpha diversity, (b) heterogeneity and alpha diversity, (c) elevation and density (individuals/ha), and (d) heterogeneity and density (individuals/ha) along an altitudinal gradient in the Dry Tropical Andes of northern Chile**.

**Fig 4:**
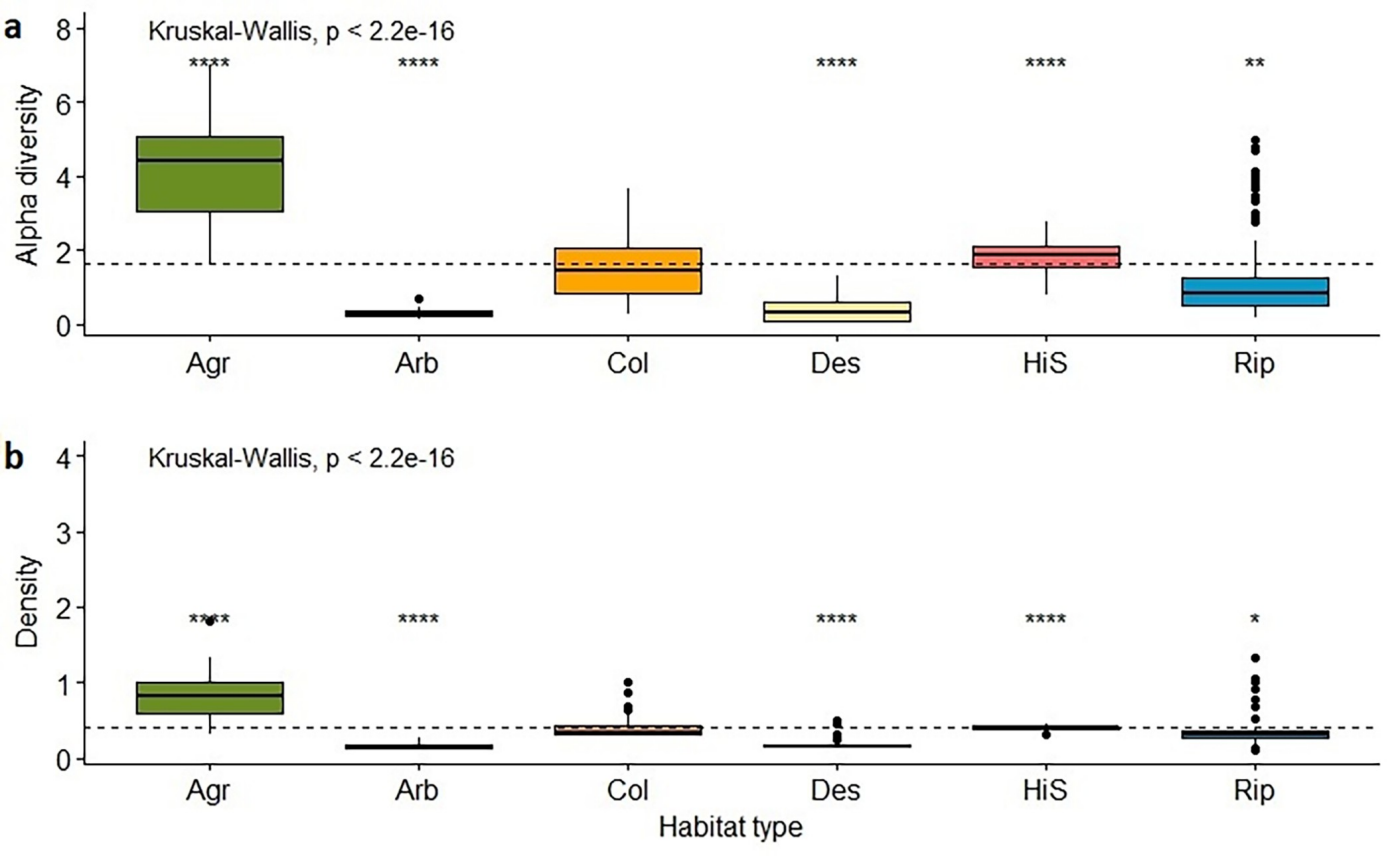
(a) Alpha diversity by habitat type and (b) bird density (individuals/ha) by habitat type, along an elevational gradient in the Dry Tropical Andes of northern Chile. Agr: Agricultural habitat; Arb: Arboreal shrubland habitat; Col: Columnar cactus habitat; Des: Desert habitat; HiS: Highland steppe habitat; Rip: Riparian habitat. Dotted lines indicate mean alpha diversity and mean density, respectively. Significant codes: <0.0001 ‘****’; <0.001 ‘***’; <0.01 ‘**’.

The pattern of alpha diversity along the elevational gradient was explained by a polynomial regression (*p*<0.05, r^2^ = 0.46, *y* = -8.9x^3^-8.8x^2^+14.6x+1.6; Fig 3A), in which increasing diversity was observed with increasing elevation up to 3500 masl (peak values of diversity), and then decreased. Alpha diversity varied from season to season, showing an increment in the wet season (0.20 [SE ± 0.007]; *p*<0.05). Vegetation heterogeneity also showed a positive association with alpha diversity (0.10 [SE ± 0.02]; *p*<0.05, r^2^ = 0.57, *Y* = 2.1x^3^ + 3.1 x^2^ + 20.9x + 1.6; Fig 3B). Finally, we found a positive association between alpha diversity and agricultural habitat (0.43 [SE ± 0.13]; *p*<0.05). Alpha diversity varied among habitat types (Kruskal-Wallis test; H = 234.8, d.f. = 5, *p*<0.05; Fig 4A), with values being relatively higher in agricultural and highland steppe habitats. By contrast, desert, riparian and arboreal shrublands showed relatively low values of alpha diversity (Wilcoxon post-hoc test *p*<0.05).

An increase in alpha diversity by elevational interval was observed in lowlands (<2500 masl). The lowest values of alpha diversity were found in the desert habitat, between 1800 and 1950 masl, with 0.09 (SE ± 0.004) species per interval (Table 3). In the interval between 1950 and 2100 masl–the riparian and arboreal shrubland habitat types–alpha diversity increased, reaching a value of 0.90 (SE ± 0.07) species per interval.

**Table 3:** Environmental characteristics, alpha diversity, beta diversity and estimated density for 19 elevational intervals (118 surveys points), surveyed between November 2016 and August 2017, in the Dry Tropical Andes of northern Chile.

Elevational interval	Elevational belt	Habitat type^(a)^	Vegetation heterogeneity (±SE)	No. of sites	Species richness^(b)^	Alpha diversity^(c)^ (±SE)	Density^(d)^ (±SE)	
**1200–1350**	Desert	Rip	6.57 (0.30)	7	5	0.45 (0.02)	0.33 (0.01)	
**1500–1650**	Desert	Rip	4.43 (1.23)	7	12	0.59 (0.04)	0.29 (0.01)	
**1650–1800**	Desert	Des/Rip	3.57 (1.60)	7	13	0.74 (0.04)	0.21 (0.01)	
**1800–1950**	Desert	Des	0.00	7	0	0.09 (0.004)	0.16 (0.002)	
**1950–2100**	Desert	Rip/Arb	4.71 (0.75)	7	13	0.90 (0.07)	0.29 (0.02)	
**2100–2250**	Desert	Rip/Arb	4.43 (0.78)	7	14	0.85 (0.06)	0.26 (0.01)	
**2250–2400**	Desert	Col	2.17 (0.98)	6	5	0.52 (0.09)	0.33 (0.02)	
**2400–2550**	Desert	Col	2.00 (0.41)	4	7	0.78 (0.05)	0.33 (0.01)	
**2550–2700**	Pre-Puna	Col	4.00 (0.22)	7	10	1.08 (0.04)	0.32 (0.004)	
**2700–2850**	Pre-Puna	Col/Agr	8.14 (1.26)	7	27	3.09 (0.19)	0.60 (0.08)	
**2850–3000**	Pre-Puna	Agr/Rip	10.29 (1.43)	7	32	4.64 (0.15)	0.76 (0.08)	
**3000–3150**	Pre-Puna	Rip/Agr	10.43 (1.29)	7	26	2.90 (0.17)	0.71 (0.05)	
**3150–3300**	Pre-Puna	Agr/Col	12.43 (0.81)	7	28	2.70 (0.26)	0.82 (0.05)	
**3300–3450**	Puna	Col	9.29 (1.04)	7	23	2.02 (0.09)	0.41 (0.02)	
**3450–3600**	Puna	HiS	7.75 (0.95)	4	17	2.04 (0.09)	0.41 (0.01)	
**3600–3750**	Puna	HiS	6.33 (1.20)	3	12	1.34 (0.07)	0.39 (0.01)	
**3750–3900**	Puna	HiS	6.67 (0.33)	3	15	2.35 (0.08)	0.39 (0.01)	
**3900–4050**	High- Andean	HiS	6.29 (0.29)	7	12	1.63 (0.06)	0.40 (0.01)	
**4050–4200**	High- Andean	HiS	6.86 (0.40)	7	14	(0.06)	0.39 (0.01)	

^(a)^Des: Desert habitat; Rip: Riparian habitat; Arb: Arboreal shrubland habitat; Col: Columnar cactus; Agr: Agricultural use habitat; HiS: Highland steppe habitat.

^(b)^Observed species richness by elevational interval.

^(c)^Estimated species richness by elevational interval.

^(d)^Individuals per hectare by elevational interval.

Alpha diversity increased from 1.08 (SE ± 0.04) species per interval at the midlands Pre-Puna belt (>2500 masl) to 4.64 (SE ± 0.15) species at the 2850–3000 masl interval. These peak values were recorded for riparian habitats and for areas with permanent presence of indigenous Aymara agriculture. Alpha diversity began to decrease with elevation, from 2.02 (SE ± 0.09) species per interval in columnar cactus habitats to 1.34 (SE ± 0.07) species per interval in highland steppe habitats in the Puna belt. In contrast, the High-Andean belt showed a higher alpha diversity than the Puna belt, reaching 1.94 (SE ± 0.06) species between 4050 and 4200 masl.

### Beta diversity along the altitudinal gradient

**Fig 5:**
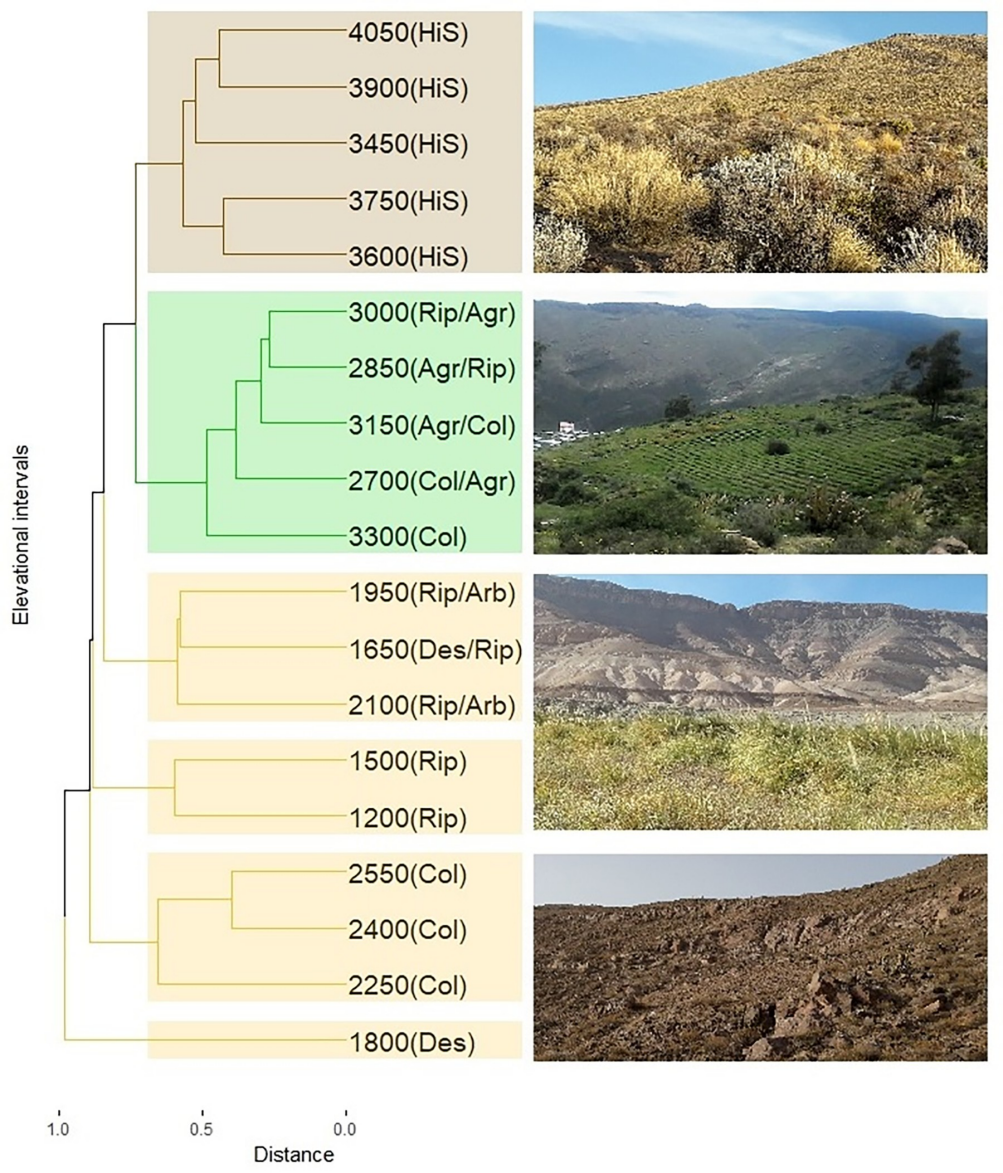
Cluster analyses based on the composition (presence/absence) of bird species across 19 elevational intervals in the northern Andes of Chile, using the Sørensen index of dissimilarity and the Unweighted Pair-Group Method (UPGMA). The yellow clusters indicate desert belt intervals, the green cluster indicates Pre-Puna belt intervals, and the brown cluster indicates Puna and High Andean belts intervals. Agr: Agricultural habitat; Arb: Arboreal shrubland habitat; Col: Columnar cactus habitat; Des: Desert habitat; HiS: Highland steppe habitat; Rip: Riparian habitat.

Beta diversity along the elevational gradient showed a relatively high turnover in species composition in lower and higher intervals (Fig 5). The most marked shift in species occurred in the lowland Desert belt (1200–2700 masl) between the different habitat types. Species composition recorded in the absolute desert habitat (interval 1800–1950 masl) was distinct from all the other habitats. In this belt, columnar cactus, riparian, and riparian with arboreal habitat types formed three different clusters of species. In the midland Pre-Puna belt, species occurring between 2700 and 3450 masl formed a distinct cluster dominated by riparian and agricultural habitat types, with columnar cactus habitat at its higher and lower limits. A final cluster of species was recorded at the highest elevations of the gradient (>3450 masl), specifically in highland steppe habitats located in the Puna and High Andean belts.

### Species density

The detectability of 17 out of 21 species analyzed in more detail was associated with survey-specific (weather or temporal) covariates (S2 Appendix). Relatively low temperature conditions (TEM) were positively associated with the detectability of nine species. By contrast, *Asthenes dorbignyi*, *A*. *modesta* and *Ashenes pubidunda* were positively associated with relatively high temperature conditions. The detectability of *M*. *aymara*, *Phrygilus plebejus* and *X*. *concolor* was positively associated with the wet season (SEA). Only *Metropelia maculirostris*, *Conirostrum cinereum* and *Phrygilus atriceps* detection rates was negatively associated with humidity (HUM).

Estimated bird density showed significant variations between elevational intervals (Kruskal-Wallis test; H = 328.2, d.f. = 18, *p*<0.05). Even so, species density did not show a strong association with elevation (*p*<0.05, r^2^ = 0.33, *y* = -2.1x^3^–1.5 x^2^ + 1.9x + 0.4; Fig 3B). No significant association between elevation and any bird species density was observed.

Density along the gradient was related to vegetation heterogeneity (HET; *p*<0.05, r^2^ = 0.63, *y* = 1.4 x^2^ + 4.2x + 0.4; Fig 4B). Overall, 11 (52.4%) species were associated with vegetation heterogeneity. Of these, 10 were positively associated with heterogeneity, with *Spinus uropygialis* (0.2 [SE ± 0.05]) and *Pipraeidea bonariensis* (0.2 [SE ± 0.04]) showing the highest slopes for the beta coefficient. Only *Sicalis uropygialis* (-0.36 [SE ± 0.05]) was negatively associated with vegetation heterogeneity.

Species density showed important differences between habitat types (Kruskal-Wallis test; H = 241.2, d.f. = 5, *p*<0.05; Fig 4B). Bird density was higher in agricultural and highland steppe habitats. In arboreal shrublands, desert and riparian habitats, density was lower compared to other habitat types (Wilcoxon post-hoc test *p*<0.05). At the species level, 15 (71.4%) were strongly associated with one or more habitat types (HAB). *P*. *plebejus* (1.69 [SE ± 0.4]) was positively associated with highland steppe habitats, while *S*. *uropygialis* (1.64 [0.3]) and *Sicalis olivacens* (0.72 [SE ± 0.2]) were associated with columnar cactus habitat.

In the lowland Desert belt (<2500 masl), maximum bird density was between 1200 and 1350 masl, with 0.33 (SE ± 0.01) individuals per hectare in the riparian habitat type. At higher elevation, the lowest density values were estimated between 1800 and 1950 masl, reaching 0.16 (SE ± 0.002) in the desert habitat type. In the midlands (2500–3300 masl), bird density increased from 0.32 (SE ± 0.004) individuals/ha between 2550 and 2700 masl to a peak of 0.82 (SE ± 0.05) individuals/ha between 3150 and 3300 masl. Above 3300 masl, bird density decreased again, reaching 0.41 (SE ± 0.02) individuals/ha at the highest elevations (Table 3).

## Discussion

This study reveals important variations in bird diversity along an elevational gradient in the Dry Tropical Andes of northern Chile, chiefly according to habitat type and vegetation heterogeneity. Existing local features at mid-elevations–such as traditional indigenous agriculture, which generates relatively high vegetation heterogeneity–may play a major role in structuring diversity and density. Human activity modifies biotic and abiotic factors, and anthropogenically constructed niches result in cascade effects that permeate the entire ecological community [6,25,74,75].

In contrast to Stevens’s rule [7], our analysis of elevational range size showed that birds with the broadest elevational ranges were more common at intermediate elevations. This finding is similar to that of a study in the Wet Tropical Andes and in the Himalayas, where a combination of biological and habitat-structural factors played a determining role in the pattern of bird elevation ranges [18,34]. In our study, 61% of the species detected showed ranges broader than 1000 m, with peaks in alpha diversity in both agricultural and riparian habitats, suggesting that these mid-elevation habitats act as a source of species that utilize a broad elevational range [15]. One possible explanation for this pattern is the mass effect hypothesis [76], in which dispersal from a more suitable habitat–in this case, from agricultural and riparian areas at mid-elevations–may add species into a less suitable habitat [41]. This is consistent with Quintero and Jetz [20], who suggest that high-elevation mountains harbor a great variety of habitats, and thus offer many opportunities for bird mixing and diversification.

On a regional scale, species turnover along the elevational gradient may be influenced by historical immigration processes in the Andes. Previous studies have suggested that bird diversity in mountain ecosystems of northern Chile was enriched by the flow of species from adjacent regions (e.g. Wet Tropical Andes and Southern Andes) [1,77–80]. These species from neighboring regions have most probably settled to specific habitat conditions along the elevational gradient, resulting in high species turnover between habitats [76,81,82].

### Elevational patterns along the gradient

The relationship between elevation and both alpha diversity and density is similar to the “hump-shaped” pattern found in other studies on plants [54,83], herpetofauna [84,85], birds [18,55,86] and small mammals [87,88]. This type of elevational pattern is characteristic of dry-based mountains [14,15], where elevation often shows a non-linear association with diversity and density [6]. Although our results showed shifts in alpha diversity and density along the elevational gradient, there was not a strong association between these parameters and elevation.

The “hump-shaped” pattern found in our study also supports the results of studies on bird diversity from other regions. For example, in the Himalayas, bird species richness and density at mid-elevations were positively associated with vegetation productivity, habitat diversity [18,55] and human settlements [89]. In the Southern Alps, the peak of bird richness at mid-elevations was attributed to optimal climatic conditions, environmental heterogeneity and man-made habitats [54]. By contrast, in the Andean forests of Bolivia, bird diversity decreased with elevation and the presence of human settlements [36]. Kessler et al. [37] showed that bird diversity was favored by the presence of old-growth forest remnants at intermediate elevations. A similar finding was reported for the Andes of Colombia, where decreasing and unimodal patterns of bird diversity were influenced by productivity [15]. For northern Chile, the unimodal pattern of terrestrial bird species was initially proposed by Vilina and Cofré [90], highlighting a diversity peak at mid-elevations. However, these authors did not explore the influence of environmental and/or anthropogenic factors on bird diversity. For their part, Gantz et al. [41] showed that peaks of bird diversity in the Atacama Desert depend on food availability and proximity to source habitats with crop vegetation. The higher number of species (n = 80) reported in the latter study compared to that of the present study (n = 49) may be explained by the former’s larger study area, as well as additional habitat types surveyed, such as littoral desert and highland wetlands.

### Habitat type and the role of traditional indigenous agriculture

Habitat type and indigenous use of valley bottoms and slopes may be strong drivers of alpha diversity and density along elevational gradients in the Andes [24,31,91]. In our study, alpha diversity was positively associated with agricultural habitats, which also showed maximum values of vegetation heterogeneity. Meanwhile, bird density only showed a positive association with agricultural habitat. Historically, the Pre-Puna belt has been one of the zones preferred for Aymara agricultural activities [32,48,49]. The positive association between agricultural habitat and bird diversity and density highlights the potential–and perhaps historical–role of traditional indigenous agriculture in mountain biodiversity. Agricultural activities provide habitats with relatively high vegetation heterogeneity, creating food and shelter resources along the elevational gradient [92]. The expansion and intensification of agriculture, along with the loss of traditional land-use in the Andes, may be the cause of rapid decline in local biodiversity [91,93–95].

In the Andes in general, the relationship of traditional mountain societies with nature has been based on coexistence rather than competition [48,96]. This relationship results in agricultural strategies based on low transformation of local geographies and resources, and thus the sustainable use of natural resources [24,96,97]. For example, practices such as the construction of a network of terraces that prevent erosion and maximize water availability, and the maintenance of unmanaged open areas for livestock grazing, allow for the growth of both native and agricultural species in an ecotone. These ecotones likely increase the diversity and abundance of food [98,99], as well as the likelihood for birds to nest close to reliable foraging habitats [100], and also address the multiple temporal requirements of species in terms of seasonal differences in vegetation phenology [101,102]. The notion that traditional Aymara agriculture has positive effects on bird diversity should be treated with caution. Our observational study was conducted along the length of a drainage basin with a particular set of socio-political and ecological characteristics, in which we found small-scale agricultural patches and communal management of streams. Future studies should implement experimental or pseudo-experimental approaches to define whether indigenous agricultural habitats have imposed an adaptive advantage for birds that utilize them.

Traditional agricultural habitats located in the midst of an arid region may support species that otherwise would not be present. For example, Norfolk et al, [103] reported that traditional agriculture supports a higher proportion of migratory and insectivorous species, and a greater number of birds associated with unmanaged habitats in the arid mountains of South Sinai. In line with this, we found *Conirostrum tamarugense* using Pre-Puna habitats during the non-breading season, possibly depending on arthropods found on cultivated and native vegetation [104]. This threatened endemic insectivore breeds in lowlands, and its activity during the winter (non-breeding) season is poorly known [104–107]. In addition, hummingbirds such as *C*. *coruscans* and *Patagona gigas peruviana* may take advantage of the winter flowering season of native and non-native species in agricultural habitats [105,108,109]. Both species are common residents in the Wet Tropical Andes [106,108,110,111] and have been rarely recorded in Chile [112–116]. Our results support those of Montaño-Centellas and Garitano-Zavala [36], who suggest that ornamental and other cultivated plants may provide foraging habitat for nectarivorous birds in the Wet Tropical Andes. This may also occur in the Dry Tropical Andes.

### Vegetation heterogeneity and seasonality

In our study, peaks in vegetation heterogeneity were associated with riparian and agricultural habitats at mid-elevations. Vegetation heterogeneity is often correlated with bird diversity [86,117–119], because heterogeneous vegetation offers more potential niches than homogeneous habitats [58,76,120]. Heterogeneity increases food and foraging opportunities [2,4], shelter and nesting substrates, and other conditions suitable for successful reproduction [121]. Availability of highly heterogeneous vegetation in Pre-Puna intervals, resulting chiefly from the presence of agricultural habitats, may explain the fact that the highest level of species similarity was observed in this belt. This high degree of similarity may be the result of a greater number of species coexisting in communities with larger niche hyperspace, causing an increase in alpha diversity and density, and a decrease in turnover [76].

The combined effect of optimal local features and climatic conditions may cause productivity to peak at mid-elevations [6]. In arid-based mountains (e.g. Dry Tropical Andes and South-Western US mountains), water availability is high at mid-elevations because rainfall and soil water retention are high, while evaporation is relatively low. In our study area, water availability decreases severely towards the lowlands–becoming concentrated in a narrow stream–where high temperature and near-absent rainfall produce extremely dry habitats. Water availability is also low in highland areas, with runoff increasing due to shallow soils and exposed rock towards mountaintops [14,43]. The presence of habitats with intrazonal vegetation in the Puna and High Andean belts, such as highland wetlands, would increase the diversity and density of birds in these elevational intervals [122–124]. However, our study basin does not comprise high-elevation wetlands. Basins with a presence of high-elevation wetlands may show a different pattern of bird diversity along the elevation gradient, as these habitats are known to be species-rich systems.

Seasonality was influential on alpha diversity, but not for species density. Only the detection rates of the granivorous *M*. *aymara* and *P*. *plebejus*, and the insectivore *X*. *concolor* were positively associated with the wet season. Studies in the Atacama Desert found that temporal variation in the diversity and density of granivorous birds may relate to variations in the supply of feeding resources determined by tropical rainfall and increases in primary productivity in northern Chile [41,125].

### Implications for conservation

Bird diversity patterns along elevational gradients should not be attributed to a single universal explanation, but rather to a combination of “natural” and anthropogenic factors [54,95]. Association among species turnover and habitat diversity along the gradient, suggesting that conservation efforts should consider the whole gradient rather than just portions of it [1,18,24,89]. Traditional agricultural habitats at mid-elevations enhanced the vegetation heterogeneity that likely provided resources for resident and rare species throughout the year.

Historical and contemporary socio-economic changes in indigenous livelihoods can potentially drive changes in “anthropogenic habitats”, and thus in bird species assemblages in the Pre-Puna belt [24,126]. Traditional Aymara agriculture is an enduring cultural practice, but has gradually been modified due to the influence of State policies, industrial development, and growth of neighboring urban centers [95,97]. Many Aymara farmers who once subsisted on what they cultivated or traded with highland camelid pastoralists have become increasingly involved in a market economy that has greatly expanded their patterns of consumption. For example, many have shifted from small-scale traditional to intensive agriculture, increasing the use of agro-chemicals that can potentially affect the value of agricultural areas as critical habitats for birds [127–129].

Despite the relatively high number of species with restricted distributions in the Dry Tropical Andes, only *C*. *tamarugense* has been assigned to a conservation category [104,130]. Several of the species detected in small numbers by our study are largely unknown in the Dry Tropical Andes, making it difficult to estimate whether our records correspond to vagrants, migratory individuals or a breeding population [113,116]. Furthermore, Pre-Puna habitats of high ecological and cultural value are among the most poorly represented elevation belts in the National System of Protected Areas of Chile (SNASPE) [131]. This situation is made worse by the fact that the mountains of northern Chile are increasingly subject to commercial interests in the form of intensive agriculture, road construction and mining operations [132–135]. Bird species occurring in the Dry Tropical Andes seem to be well adapted to their local environments; however, some of these species may represent the last stage in a taxon cycle [1], surviving as local remnant populations in their southernmost distribution range.

## Supporting information

S1 Appendix Birds observed along an elevational gradient in the Dry Tropical Andes of northern Chile, surveyed in wet (W) and dry (D) seasons between November 2016 and August 2017. (DOCX)

S2 Appendix Bird species and covariates^(a)^ used to estimate detectability (*p*) and density (*D*) in the elevational gradient, according to the selection of models based on the Akaike’s Information Criterion (AIC). Positive (+) and negative (-) symbols indicate the direction of the relationship; values in parentheses ( ) indicate the standard error. See “S1 Appendix” for codes of bird species.
(DOCX)
