# Supplementary material for: Bird diversity along elevational gradients in the Dry Tropical Andes of northern Chile: The potential role of Aymara indigenous traditional agriculture
Source: PLoS One. 2018 Dec 5;13(12):e0207544. doi: 10.1371/journal.pone.0207544 (PMC6281285; doi:10.1371/journal.pone.0207544)
Supplement: S1 Appendix — (DOCX) [file pone.0207544.s001.docx]

**S1 Appendix. Birds observed along an elevational gradient in the Dry Tropical Andes of northern Chile, surveyed in wet (W) and dry (D) season between November 2016 and August 2017.**

| Species | English name | Code | Feb  W | Apr  W | Aug  D | Nov  D |
| --- | --- | --- | --- | --- | --- | --- |
| *Patagioenas maculosa albipennis* | Spot-winged Pigeon | PATMAC | X |  | X | X |
| *Zenaida meloda* | West Peruvian Dove | ZENMEL |  | X |  |  |
| *Metriopelia ceciliae* | Bare-faced Ground Dove | METCEC | X |  | X | X |
| *Metriopelia melanoptera* | Black-winged Ground Dove | METMEL | X | X | X | X |
| *Metriopelia aymara* | Golden-spotted Ground Dove | METAYM | X | X | X | X |
| *Aeronautes andecolus* | Andean Swift | AERAND | X | X |  | X |
| *Colibri coruscans* | Sparkling Violetear | COLCOR |  | X | X | X |
| *Oreotrochilus estella* | Andean Hillstar | OREEST | X | X | X | X |
| *Patagonas gigas peruviana* | Giant Hummingbird | PATGIG |  | X | X |  |
| *Rhodopis vesper* | Oasis Hummingbird | RHOVES | X | X | X | X |
| *Thinocorus orbignyanus* | Gray-breasted Seedsnipe | THIORB |  |  |  | X |
| *Thinocorus rumicivorus* | Least Seedsnipe | THIRUM |  |  |  | X |
| *Vultur gryphus* | Andean condor | VULGRY |  |  | X |  |
| *Elanus leucurus* | White-tailed Kite | ELELEU |  | X |  |  |
| *Geranoaetus polyosoma* | Variable Hawk | GERPOL |  | X | X | X |
| *Phalcoboenus megalopterus* | Mountain caracara | PHAMEG |  |  | X |  |
| *Falco sparverius* | American kestrel | FALSPA | X | X |  | X |
| *Falco femoralis* | Aplomado Falcon | FALFEM | X | X |  |  |
| *Geositta punensis* | Puna Miner | GEOPUN | X |  |  |  |
| *Ochetorhynchus ruficaudus* | Straight-billed Earthcreeper | OCHRUF | X | X | X | X |
| *Phleocryptes melanops* | Wren-like Rushbird | PHLMEL | X | X |  |  |
| *Cinclodes atacamensis* | White-winged Cinclodes | CINATA |  | X |  |  |
| *Leptasthenura aegitaloides* | Plain-mantled Tit-Spinetail | LEPAEG | X | X | X | X |
| *Asthenes dorbignyi* | Creamy-breasted Canastero | ASTDOR | X | X | X | X |
| *Asthenes modesta* | Cordilleran canastero | ASTMOD | X | X | X | X |
| *Asthenes pubidunda* | Canyon Canastero | ASTPUB | X | X | X | X |
| *Elaenia albiceps* | White-crested Elaenia | ELAALB | X |  |  |  |
| *Anairetes flavirostris* | Yellow-billed Tit-Tyrant | ANAFLA | X | X | X | X |
| *Pyrocephalus rubinus* | Vermilion Flycatcher | PYRRUB |  |  |  | X |
| *Muscisaxicola maculirostris* | Spot-billed Ground-Tyrant | MUSMAC | X | X | X |  |
| *Muscisaxicola cinereus* | Cinereous Ground-Tyrant | MUSCIN |  | X |  |  |
| *Muscisaxicola rufivertex* | Rufous-naped Ground-Tyrant | MUSRUF |  | X | X |  |
| *Muscisaxicola maclovianus* | Dark-faced Ground-Tyrant | MUSMACL |  | X | X |  |
| *Ochthoeca leucophrys* | White-browed Chat-Tyrant | OCHLEU | X | X | X |  |
| *Tyrannus melancholicus* | Tropical Kingbird | TYRMEL |  | X |  | X |
| *Orochelidon andecola* | Blue-and-white Swallow | OROAND | X | X | X | X |
| *Turdus chiguanco* | Chiguanco Thrush | TURCHI | X | X | X | X |
| *Conirostrum tamarugense* | Tamarugo Conebill | CONTAM | X | X |  |  |
| *Conirostrum cinereum* | Cinereus Conebill | CONCIN | X | X |  | X |
| *Pipraeidea bonariensis* | Blue-and-yellow Tanager | PIPBON | X | X | X | X |
| *Phrygilus atriceps* | Black-hooded Sierra-Finch | PHRATR | X | X | X | X |
| *Phrygilus fruticeti* | Mourning Sierra-Finch | PHRFRU | X | X | X | X |
| *Phrygilus plebejus* | Ash-breasted Sierra Finch | PHRPLE | X | X | X | X |
| *Sicalis uropygialis* | Bright-rumped Yellow-Finch | SICURO | X | X | X | X |
| *Sicalis olivascens* | Greenish Yellow-Finch | SICOLI | X | X | X | X |
| *Xenospingus concolor* | Slender-billed Finch | XENCON | X | X | X | X |
| *Zonotrichia capensis* | Rufous-collared Sparrow | ZONCAP | X | X | X | X |
| *Spinus magellanicus* | Hooded Siskin | SPIMAG | X | X | X | X |
| *Spinus uropygialis* | Yellow-rumped Siskin | SPIURO | X | X | X | X |
