# Supplementary material for: Bird diversity along elevational gradients in the Dry Tropical Andes of northern Chile: The potential role of Aymara indigenous traditional agriculture
Source: PLoS One. 2018 Dec 5;13(12):e0207544. doi: 10.1371/journal.pone.0207544 (PMC6281285; doi:10.1371/journal.pone.0207544)
Supplement: S2 Appendix — Positive (+) and negative (-) symbols indicate the direction of the relationship; values in parentheses ( ) indicate the standard error. See “S1 Appendix” for codes of bird species. (DOCX) [file pone.0207544.s002.docx]

**S2 Appendix**. **Bird species and covariates^(a)^ used to estimate detectability (*p*) and density (*D*) in the elevational gradient, according to the selection of models based on the Akaike’s Information Criterion (AIC).** Positive (+) and negative (-) symbols indicate the direction of the relationship; values in parentheses ( ) indicate the standard error. See “Appendix S1” for codes of bird species.

|  | Detectability (*p*) | | | | | | | | | Density (*D*) | | | | | | |
| --- | --- | --- | --- | --- | --- | --- | --- | --- | --- | --- | --- | --- | --- | --- | --- | --- |
|  | **DAT** | **TIM** | **WIN** | **TEM** | **NOI** | | **SEA** | | **HUM** | **HAB** | | | | | | **HET** |
|  |  |  |  |  | **0** | **1** | **Dry** | **Wet** |  | **Des** | **Arb** | **Col** | **Agr** | **Rip** | **HiS** |  |
| METAYM | 0.007 (0.003) |  |  |  |  |  |  | 2.24 (0.86) |  |  |  |  |  |  |  |  |
| AERAND |  | 0.13 (0.007) |  |  | -2.74 (0.35) | 5.58 (0.23) |  | -2.22 (0.35) |  | -1.1 (0.48) |  | -7.2 (2.2) |  | -3.08 (0.69) |  | 0.16 (0.05) |
| RHOVES |  |  |  |  |  |  |  |  |  |  |  | -2.03 (0.67) |  | -2.46 (0.75) |  |  |
| OCHRUF |  | -0.006 (0.002) |  |  |  |  |  |  |  |  |  |  |  |  |  |  |
| LEPAEG |  |  | 1.1 (0.03) | -0.07 (0.02) |  |  |  |  |  |  |  | -1.77 (0.62) |  | -1.67 (0.67) |  |  |
| ASTDOR |  |  |  | 1.24 (0.004) | -8.28 (0.006) |  |  |  |  |  |  |  |  |  |  | 0.13 (0.05) |
| ASTMOD |  |  |  | 1.09 (0.003) | -1.16 (0.004) | -4.6 (0.78) |  |  |  |  |  |  |  |  |  |  |
| ASTPUB |  |  |  | 1.06 (0.03) |  |  |  |  | 0.1 (0.2) |  |  | -1.76 (0.65) | -2.99 (0.8) |  |  | 0.18 (0.06) |
| MUSMAC |  |  | 0.19 (0.003) |  |  |  |  |  | -0.09 (0.03) |  |  |  |  |  |  | 0.14 (0.06) |
| TURCHI |  |  |  | -0.08 (0.04) |  |  |  |  |  |  |  | -2.48 (0.63) |  |  |  |  |
| CONCIN |  |  |  | -1.1 (0.02) |  |  |  |  | -0.05 (0.02) |  |  | -1.94 (0.6) |  | -1.68 (0.58) |  |  |
| PIPBON |  | -0.007 (0.002) |  | 0.08 (0.03) | 2.68 (0.29) |  |  |  |  |  |  | -1.44 (0.39) | -1.57 (0.6) | -1.39 (0.4) |  | 0.2 (0.04) |
| PHRATR |  |  |  | -0.7 (0.03) |  |  |  |  | -0.32 (0.03) |  |  |  | -2.78 (0.91) |  |  |  |
| PHRFRU |  |  |  |  |  |  |  |  |  |  |  | -1.44 (0.3) | -0.98 (0.34) | -1.08 (0.29) | -2.48 (0.3) | 0.18 (0.02) |
| PHRPLE |  |  |  | -0.05 (0.2) |  |  |  | 1.03 (0.31) |  |  |  |  | -1.01 (0.4) |  | 1.69 (0.4) |  |
| SICURO |  | -0.04 (0.004) |  | -0.3 (0.34) |  |  |  |  |  |  |  | 1.64 (0.28) |  |  |  | -0.36 (0.05) |
| SICOLI | 0.002 (0.0005) |  |  | -0.05 (0.01) |  |  |  |  |  |  |  | 0.72 (0.24) |  |  |  | 0.06 (0.02) |
| XENCON |  |  |  |  |  |  |  | 1.97 (0.72) | 0.02 (0.01) |  |  |  |  |  |  |  |
| ZONCAP |  |  |  |  |  |  |  |  |  | 0.85 (0.37) |  | -1.53 (0.39) | -1.66 (0.42) |  | -3.36 (1.27) | 0.18 (0.03) |
| SPIMAG |  |  |  | -0.27 (0.02) |  |  |  |  |  | -3.81 (0.01) | -2.98 (1.01) | -2.46 (0.30) | -1.7 (0.34) | -1.98 (0.28) | -2.1 (0.26) | 0.17 (0.02) |
| SPIURO |  |  |  | -0.08 (0.02) | 4.18 (0.35) | -5.58 (57.47) |  |  |  |  |  |  |  |  | -2.9 (0.8) | 0.2 (0.05) |

1. Covariates; DAT: date; TIM: time; WIN: wind velocity; TEM: temperature; NOI: noise; SEA: season; HUM: humidity; HAB: habitat type; HET: heterogeneity; Agr: Agricultural habitat; Arb: Arboreal shrubland habitat; Col: Columnar cactus habitat; Des: Desert habitat; HiS: Highland steppe habitat; Rip: Riparian habitat.
